# The C-type lectin AcCTL5 from *Apis cerana* mediates anti-*Nosema ceranae* defense via agglutination and hemolymph melanization

**DOI:** 10.1080/21505594.2026.2687914

**Published:** 2026-06-10

**Authors:** Dufu Li, Tingyue Huang, Qianmin Hai, Qiang Ma, Xuelu Liu, Xiaoqun Dang, Zhengang Ma, Jinshan Xu, Zeyang Zhou

**Affiliations:** a Key Laboratory of Pollinator Resources Conservation and Utilization of the Upper Yangtze River, Ministry of Agriculture and Rural Affairs, Chongqing Normal University, Chongqing, China; b Chongqing Key Laboratory of Vector Control and Utilization, College of Life Sciences, Chongqing Normal University, Chongqing, China; c Department of Basic Medicine, Chongqing Three Gorges Medical College, Chongqing, China

**Keywords:** CTL5, *Apis cerana*, *Nosema ceranae*, biological function, immune

## Abstract

Unlike vertebrates, which use adaptive immunity, insects depend on humoral and cellular innate immunity to resist pathogens, with pattern recognition receptors (PRRs) like C-type lectins (CTLs) critical for recognizing pathogen-associated molecular patterns (PAMPs). CTLs are a diverse, mostly Ca^2+^-dependent glycoprotein superfamily defined by conserved Carbohydrate Recognition Domains (CRDs), enabling ligand binding and mediating immune defenses in invertebrates. *Apis cerana*, a vital pollinator, faces severe threats from pathogens such as *Nosema ceranae*, a major cause of nosemosis that damages midguts, reduces vitality, and triggers colony collapse. This highlights the urgency of studying bee immune mechanisms. Here, we identified AcCTL5 (a CTL from *A. cerana*), prepared, purified its recombinant protein, and characterized its functions. Recombinant AcCTL5 strongly agglutinated *N. ceranae*, bound significantly to PAMPs (galactose, peptidoglycan, lipoteichoic acid, lipopolysaccharide), reduced honeybee infection rates, and participated in prophenoloxidase activation and hemolymph melanization. These results suggested that *AcCTL5* may play an important role in the immune defense response of *A. cerana* against *N. ceranae*, and lay the foundation for further research on the molecular mechanism of *AcCTL5* involved in the innate immunity of *A. cerana*.

## Introduction

Vertebrates rely on an efficient adaptive immune system to adapt to complex and changing living environments, while insects mainly rely on innate immunity, composed of humoral immunity and cellular immunity, to resist the invasion of pathogenic microorganisms in the living environment [1,2]. Humoral immunity mainly includes the production of synthetic antibacterial effectors via signal transduction pathways and the activation of the prophenoloxidase system [3,4]. Cellular immunity is mainly regulated by the circulation of hemocytes, including phagocytosis, encapsulation, melanization, and nodule formation [5]. A critical initial step in the innate immune response of insects is the recognition of invading pathogens. Pattern recognition receptors (PRRs) control this recognition process and specifically bind to conserved pathogen-associated molecular patterns (PAMPs), such as peptidoglycan (PGN), lipopolysaccharides (LPS), lipoteichoic acid (LTA), galactose (Gal), and mannose (MAN). Thereby, PRRs regulate insect humoral and cellular immunity to clear and destroy invading pathogenic bacteria [6,7].

C-type lectins (CTLs) constitute a superfamily of Ca^2+^-dependent glycoproteins widely distributed in vertebrates and invertebrates. They derive their name from their initial discovery in calmodulin and their requirement for Ca^2+^ to bind carbohydrates, and represent the most diverse and abundant family within the larger lectin superfamily [8,9]. But it has recently been shown that some CTLs can also recognize ligands without the presence of Ca^2+^ [10]. A defining hallmark of CTLs is the presence of a conserved Carbohydrate Recognition Domain (CRD), which enables the specific recognition and binding of carbohydrate molecules on the surface of pathogenic microorganisms or host cells. This structural feature underpins the pivotal roles of CTLs in key physiological processes, including immune defense, cell adhesion, and developmental regulation [11]. Each CRD typically comprises 110–130 amino acid residues and adopts a canonical bicyclic conformation. Within this domain, two highly conserved amino acid motifs—EPN (Glu-Pro-Asn) and QPD (Gln-Pro-Asn)—are integral to the specific recognition of carbohydrates. Specifically, the EPN motif primarily mediates binding to MAN, while the QPD motif plays a critical role in Gal binding. These two motifs thus serve as reliable indicators for predicting the carbohydrate-binding specificity of C-type lectins [12–15]. The CRD can specifically recognize and bind carbohydrates in the cell wall of invading pathogenic microorganisms, thereby causing the body’s immune response [16]. In addition to carbohydrates, C-type lectins can also recognize a variety of ligands, such as proteins, lipids, and inorganic molecules [17]. CTLs can be used as upstream pattern recognition receptors to recognize multiple microorganisms, as well as downstream effectors with bactericidal activity, and can also sense dead and cancerous cells [18,19]. Invertebrate C-type lectins have been shown to regulate immune responses and growth and development [20].

The CTL of insects is involved in a variety of immune responses and plays an important role in immune defense. The *Bombyx mori* multi-binding protein (BmMBP) in the hemolymph of the domestic silkworm binds to Gram-positive bacteria, Gram-negative bacteria, and yeast, thereby causing agglutination of blood cells and nodule formation [21]; BmCTL-S3 can act as an opsonin to effectively eliminate bacteria [22]; and BmCTL-S6 may serve as a PRR for recognizing foreign pathogens, stimulating the prophenoloxidase pathway, and participating in innate immunity [23]. The C-type lectins, immulectins in *Manduca sexta*, are involved in cellular immunity and humoral immunity, and regulate melanization, encapsulation, and agglutination reactions [24,25], among which immulectin-1 and -2 are involved in the activation of prophenoloxidase, and immulectin-3 and -4 enhanced melanization and encapsulation [26]. The CTL1 of *Drosophila melanogaster* is involved in the phagocytosis of blood cells and has an agglutinating effect on *Escherichia coli* [27]. The C-type lectin IML-10 of *Ostrinia furnacalis* enhances cell aggregation by directly binding to the surface of blood cells and further enhances cell encapsulation [28]. The C-type lectins HaCTL3, HaCTL4, HaCTL5, HaCTL6, HaCTL7, and HaCTL8 in *Helicoverpa armigera* are pattern recognition receptors of immune defense fungi in *H.armigera* and are mediated by 20-hydroxyecdysone (20E) for their regulation of immunity [29,30]. RNA interference-mediated knockdown of HaCTL3 was found to inhibit the ecdysone and juvenile hormone signaling pathways, resulting in reduced larval body size, delayed pupation, and increased abundance of *Enterococcus mundtii* in the hemolymph. This evidence demonstrates that the insect immune system can modulate the endocrine system to regulate development by shaping the composition of the hemolymph microbiota [31]. In the antiviral experiments on *Musca domestica*, MdCTL1-2 significantly reduced the infection rate of *Spodoptera frugiperda* 9 (sf9) cells by *Autographa californica* multicapsid nucleopolyhedrovirus (AcMNPV) [32].

As a typical representative of pollinating insects, bees are the main economic insect pollinating entomophilous plants in the world. However, during the breeding and survival of bees, they are easily threatened by various diseases such as viruses, fungi, bacteria, and microsporidia, which have brought huge economic losses to the beekeeping industry [33,34]. Among these pathogens, *N. ceranae* is one of the main causative agents of honeybee nosemosis. It infects the midgut epithelial cells of adult honeybees, thereby causing affected bees to exhibit symptoms including midgut ulceration, atrophy, reduced vitality, shortened lifespan, disrupted colony order, and even colony collapse. Furthermore, the reduced immunity induced by *N. ceranae* exposes honeybees to the risk of co-infection with other pathogens, which further accelerates colony collapse [35]. Therefore, studying the immune defense mechanism of bees is of great significance for controlling bee diseases and maintaining the long-term stability of bee farming. In this study, we have reported a member of AcCTL5 from the CTLs family of *A. cerana*. We prepared and purified recombinant AcCTL5 and studied its carbohydrate-binding specificity, agglutinating activity, and role in immune response. We found that the recombinant AcCTL5 could significantly bind to PGN, Gal, LTA, and LPS, as well as *N. ceranae*, and exhibited strong agglutinating activity. In addition, the recombinant AcCTL5 could effectively reduce the infection rate of honeybees and was involved in the activation of prophenoloxidase and the melanization of hemocytes. This study provides useful clues for the prevention and treatment of bee pathogenic diseases.

## Materials and methods

### Experimental organisms

#### 5-day-old Apis cerana

The 5-day-old *A. cerana* utilized in this study were sourced from a colony maintained at the Beekeeping Base of Chongqing Normal University (29.36◦N, 106.17◦E). *N. ceranae* was previously purified and preserved in Key Laboratory of Pollinator Resources Conservation and Utilization of the Upper Yangtze River.

### Identification and sequence analysis of CTL5

Twelve fragments with CRD structures (named AcCTL1-AcCTL12) were screened from the cDNA library of *Apis cerana*, which have similar sequences to typical C-type lectins [32]. Among these, the sequence similarity between AcCTL5 and the CTL5 of *Apis mellifera* is 100%; it is speculated that it may play an important role in the process of honeybee innate immunity. Therefore, AcCTL5 was selected as the target for further experiments to explore its immune functions. AcCTL5-specific primers were used (Table 1), according to the conditions: predenaturation at 94°C for 5 min, 25 cycles of denaturation at 94°C for 40 s, annealing at 55°C for 50 s, and extension at 72°C for 1 min. The sequence of AcCTL5 was amplified by PCR and confirmed by re-sequencing. The encoded amino acid sequence, molecular weight, and isoelectric point were predicted using the EXPASY (Expert Protein Analysis System) website (http://www.expasy.org). The presence and location of signal peptides were predicted using the SignalP 4.1 program (http://www.cbs.dtu.dk/services/SignalP4.1/). Protein domain structure prediction was performed using SMART (http://smart.emblheidelberg.de/smart/set_mode.cgi). Similarity analysis was performed using BLAST (http://www.ncbi.nlm.nih.gov/). Phylogenetic trees were reconstructed using the maximum likelihood (ML) method in MEGA 12 [36]. The LG+G model (LG with gamma-distributed rates) was used for amino acid substitutions, and nodal support was assessed with 1000 bootstrap replicates. The tree was visualized using the online iTOL platform.

**Table 1.** Oligonucleotide primers for this study.

Gene name	Primer name	Primer sequence	PCR length
AcCTL5 (for RT-q PCR and cloning)	AcCTL5_F	5’-ATATGGATCCATGAGGATGATATGGCTAAT-3’	650bp
	AcCTL5_R	5’-ATATCTCGAGAAGACGAATGCCAGGGTTTC-3’	
Actin	Actin_F	5’-TCCTGCTATGTATGTCGC-3’	200bp
	Actin_R	5’-AGTTGCCATTTCCTGTTC-3’	

### Analysis of tissue expression profile of CTL5 in A. cerana

In order to analyze the mRNA expression levels in different tissues of healthy adult *A. cerana*, seven individuals were randomly selected, and eight different tissues, namely head, chest, epidermis, midgut, fat body, hemolymph, malpighian tubules, and leg, were dissected. Tissues of the same type from the seven bees were pooled together in RNase-free centrifuge tubes, immediately frozen in liquid nitrogen, and stored at −80°C until use. The entire dissection and pooling process was performed quickly on ice to prevent the degradation of RNA.

### RNA samples preparation and cDNA synthesis

Total RNA was extracted from each pooled tissue sample using TRIzol reagent (Thermo Fisher Scientific, USA) following the manufacturer’s instructions. One μL of sample was taken, a NANO DROP 1000 nucleic acid concentration analyzer was used to measure the RNA concentration of each tissue, and the integrity of total RNA was evaluated by electrophoresis on 2% agarose gel. First‑strand cDNA was synthesized using the PrintScript™II 1st Strand cDNA Synthesis Kit (Takara, Japan). The reverse transcription reaction was performed at 25°C for 10 min, 42°C for 60 min, and 70°C for 15 min. The resulting cDNA was stored at −20°C until use. Quantitative real-time PCR (qPCR) was carried out on a Bio‑Rad CFX96 real-time PCR system (Bio‑Rad, USA) using Taq Pro Universal SYBR qPCR Master Mix (Vazyme, China). Each 20 μL reaction contained: 10 μL SYBR Master Mix, 0.4 μL each of forward and reverse primers (10 μM), 1 μL cDNA template, and 8.2 μL ddH_2_O. The thermal cycling program was: 95°C for 30 s; 40 cycles of 95°C for 10 s, 60°C for 10 s with signal acquisition, and 72°C for 30 s. The primers used for *AcCTL5* and the reference gene *AcActin* were those listed in Table 1. Primer specificity was confirmed by melting curve analysis, and a single peak was observed for each amplicon. For each tissue sample, three technical replicate reactions were performed. Relative expression levels of *AcCTL5* were calculated using the 2^−ΔΔCt^ method, with *AcActin* as the internal control.

### Recombinant expression of CTL5 in E. coli and preparation of the antibody

The Open Reading Frame (ORF) region of the AcCTL5 without signal peptide-encoding fragment was cloned using the specific primers listed in Table 1 that provided the restriction sites *BamHI* and *XhoI*. The amplified PCR products were ligated with the pMD19-T vector for sequencing verification and subcloned into the pET32 plasmid. The recombinant plasmid was verified by DNA sequencing and transformed into *E. coli* strain Rosetta (DE3) for prokaryotic expression. Cells were incubated in LB medium (containing 50 μg/mL ampicillin) at 37°C with shaking at 220 rpm to an OD_600_ = 0.6, bacteria carrying the recombinant AcCTL5 (rAcCTL5) plasmid were induced with 0.05 mM of Isopropyl-β-D-thiogalactoside (IPTG), and cultured for an additional 24 h at 16°C. Cells were harvested and expression of rAcCTL5 was assessed by SDS-PAGE. Since the expression mode of the recombinant protein can be reflected in two forms of inclusion body and soluble protein. The expression form of rAcCTL5 was determined by ultrasonic disruption of the bacterial cells. Briefly, the bacterial pellet was resuspended in PBS and subjected to sonication using an XO‑650D ultrasonicator from Nanjing Xianou Instrument Manufacturing Co., Ltd. on ice for 30 min with a 3 mm probe, 50 W output, and working cycles of 3 s on and 2 s off. The lysate was then centrifuged at 12,000 rpm for 10 min at 4°C. Aliquots of the supernatant and the resuspended pellet were mixed with 3× SDS loading buffer, heated at 100°C for 10 min, and then separated by SDS‑PAGE at 80 V for the stacking gel and 120 V for the resolving gel to assess the solubility of rAcCTL5. Soluble proteins were purified according to His affinity chromatography on Ni-NAT Superflow Cartridge (QIAGEN, Germany). Four mice were immunized with purified rAcCTL5 protein mixed with Freund’s adjuvant (Sigma, USA) at a ratio of 1:1, once every 7 days, and immunized four times in total. Polyclonal antibody serum was obtained and stored at −20°C for future use.

### Western blotting

The obtained recombinant protein was separated by 12.5% SDS-PAGE and then transferred to a polyvinylidene fluoride (PVDF) membrane. After protein transfer, they were blocked overnight at 4°C with 10 mL of TBST containing 5% nonfat dry milk. Then, the membrane was washed 3 times with TBST and incubated with mouse polyclonal anti-AcCTL antibody (1:100) at 37°C for 2 h. After incubation, the membrane was washed 3 times with TBST and incubated with horseradish peroxidase (HRP)-labeled goat anti-mouse IgG (H+L) (1:2000, Beyotime, China) at 37°C for 1 h. Finally, the detection band was incubated with Ecl substrate chromogenic solution according to the manufacturer’s instructions (Beyotime, China).

### Binding experiment of the cell wall sugar component

To determine the sugar‑binding specificity of AcCTL5, seven different types of carbohydrates were used from Sigma (USA), namely mannose, galactose, peptidoglycan, lipopolysaccharide, zymosan, laminarin, and lipoteichoic acid. Carbohydrates at a concentration of 80 μg/mL were coated on a 96-well plate, and 10 μg/mL BSA (Beyotime, China) was added for blocking at 37 °C for 2 h. The blocking solution was discarded, and the wells were incubated with 20 μg/mL rAcCTL5 protein at room temperature for 1–2 h; a control experiment was performed using 20 μg/mL Trx‑Tag protein. After washing three times with TBS, anti-His antibody (1:2000, Beyotime, China) was added and incubated at 37 °C for 2 h. The wells were then washed three times with TBS, and HRP-labeled goat anti-mouse secondary antibody (1:2000, Beyotime, China) was added and incubated at 37°C for 1 h. Finally, TMB chromogenic solution was added and the plate was incubated at 37°C in the dark for 10 min. When a blue color appeared, 2 M H_2_SO_4_ was added to stop the color reaction, and the absorbance was measured at 450 nm. The experiments were repeated three times. A difference was considered significant at *p* < 0.05.

### Immunofluorescence

*N. ceranae* at a cell density of 1.45 × 10^8^ CFU/mL was incubated with 20 μg/mL rAcCTL5 protein at 27°C for 45 min. For control experiments, Trx-Tag protein (20 μg/mL) was used instead of rAcCTL5 and processed under the same conditions. The samples were fixed with 4% paraformaldehyde at 4°C for 12 h, washed three times with PBS, and then incubated with mouse polyclonal anti-AcCTL5 antibody (1:100) at 37°C for 2 h. Anti-His antibody (1:100, Beyotime, China) was used as a negative control. After incubation, the samples were washed three times with PBS and incubated with FITC-labeled goat anti-mouse secondary antibody IgG (H+L) (1:200, Sigma, USA) for 1 h at 37 °C in the dark. Subsequently, three fields of view were randomly selected under a 100 × oil immersion lens using an Olympus DP80 fluorescence microscope (Olympus Corporation, Tokyo, Japan) to observe the binding phenomenon.

### Pathogenic microorganism agglutination assay

The direct agglutination ability of rAcCTL5 was tested with *N. ceranae*. The microorganism at a cell density of 1.45 × 10^8^ CFU/mL was incubated with 80 μL FITC solution (10 mg/mL DMSO) at 37°C for 1 h in the dark. After washing with TBS three times, it was fixed with 4% paraformaldehyde for 30 min. The FITC-labeled *N. ceranae* was suspended in 1 mL of TBS (containing 1 mM Mg^2+^, 10 mM Ca^2+^) and mixed with 20 μg/mL rAcCTL5 protein. At the same time, a control experiment was performed with 20 μg/mL Trx-Tag-tagged protein. After the mixture was incubated at room temperature (RT) in the dark for 1 h, 5 µL was pipetted onto a glass slide. After natural drying, an appropriate amount of anti-fluorescence quencher was added to seal the slide. Subsequently, three fields of view were randomly selected under a 100× oil immersion lens using an Olympus DP80 fluorescence microscope (OLYMPUS, Tokyo, Japan) to observe the agglutination phenomenon. The results were statistically analyzed based on the criteria that the diameter of the formed colonies was greater than 10 μm and the number of *N. ceranae* aggregates was more than 3.

### Antibody blocking of *N.* *ceranae*

Healthy adult *A. cerana* were randomly divided into 3 groups (10 in each group), and the effect of rAcCTL5-treated *N. ceranae* on the survival rate of honeybees was observed. The first group: was fed with 50% sucrose solution; the second group: mixed *N. ceranae* with a cell density of 1.5 × 10^4^ CFU/mL with 50% sucrose solution, and fed the bees orally; The third group: *N. ceranae* with a cell density of 1.45 × 10^8^ CFU/mL and 20 μg/mL rAcCTL5 protein were incubated for 50 min in vitro, then mixed with 50% sucrose solution, and fed to bees orally. The number of dead bees was monitored every 12 h for a total of 9 days, and the death rate of bees was calculated every day. The experiments were repeated three times. A difference was considered to be significant at *p* < 0.05.

### rAcCTL5 activation assay of phenoloxidase

Hemolymph was collected from healthy adult *A. cerana*, and blood cells were removed by centrifugation at 500 g for 5 min at 4°C. The cell-free hemolymph was used for the following two groups of experiments. For the first group, 20 µL of hemolymph and 30 µL of rAcCTL5 protein (1.5 µg) were mixed evenly and added to a 96-well plate; at the same time, 20 µg/mL Trx-Tag protein was added as a control. For the second group, 20 µL of hemolymph and 30 µL of rAcCTL5 protein (1.5 µg) were mixed evenly and added to a 96-well plate, with galactose added to the experimental group and PBS added to the control group. The OD value was then measured every 5 min at 450 nm, and the results were visualized using GraphPad Prism 5.

### Statistical analysis

Data were presented as mean ± SD. For comparisons between two groups, Student’s t‑test was used. For multiple group comparisons, one‑way analysis of variance (ANOVA) followed by Tukey’s HSD post‑hoc test was applied. For survival analysis, Kaplan‑Meier curves were generated and compared using the log‑rank test. A *p* value < 0.05 was considered statistically significant.

## Results

### CTL5 has a QPD motif and a complete CRD domain

**Figure 1.**
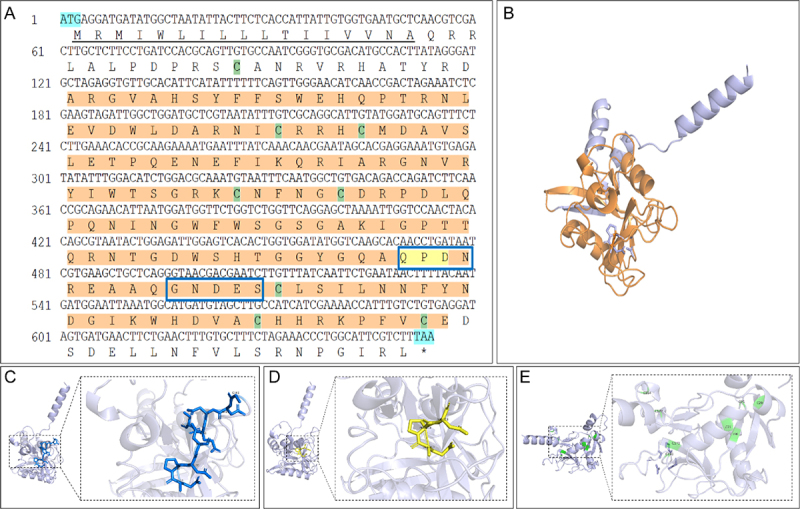
Nucleotide sequence, deduced amino acid sequence, and structural visualization of AcCTL5. (A) The numbers on the left represent the corresponding positions of the nucleotide sequence; the start codon (ATG) and stop codon (TAA) are marked in blue, and the black underlined region indicates the signal peptide. The orange shaded region is the carbohydrate recognition domain (CRD) of AcCTL5, the yellow region is the QPD (Gln-Pro-Asp, glutamine-proline-aspartic acid) motif, the green regions are the 8 conserved cysteine residues of AcCTL5, and the blue-boxed regions are the two Ca^2+^-binding sites of AcCTL5. (B) Carbohydrate recognition domain (CRD) of AcCTL5 (orange). (C) Ca^2+^-binding sites of AcCTL5 (blue). (D) QPD motif of AcCTL5 (yellow). (E) 8 conserved cysteine residues of AcCTL5 (green).

The full-length cDNA of AcCTL5 is 1195 bp, including 225 bp 5’ untranslated region (5’ UTR), 316 bp 3’ untranslated region (3’ UTR), and 654 bp open reading frame (ORF), encoding 217 amino acids. The theoretical molecular weight is 25.07 kDa, and the predicted pI is 8.41. The results of bioinformatics analysis showed that the N-terminus of the protein contained a signal peptide consisting of 17 amino acids (Figure 1, underlined), and the C-terminus contained a CRD (orange), without a transmembrane domain. Further analysis showed that there is a QPD (Gln^157^-Pro^158^-Asp^159^) motif (yellow) that can specifically bind galactose in the CRD region of AcCTL5. ScanProsite analysis showed that AcCTL5 contains 8 conserved cysteine residues (Cys^29^ and Cys^71^, Cys^75^, Cys^109^, Cys^114^, Cys^171^, Cys^190,^ and Cys^198^) (green), 4 of which are used to construct the basic skeleton of CRD, which is important for folding. Stability is critical; there are also 2 Ca^2+^ -binding sites (blue). In addition, we performed structural visualization of the CRD domain, QPD motif, 8 conserved cysteine residues, and 2 Ca^2+^ -binding sites of AcCTL5, and labeled their key sites.

**Figure 2.**
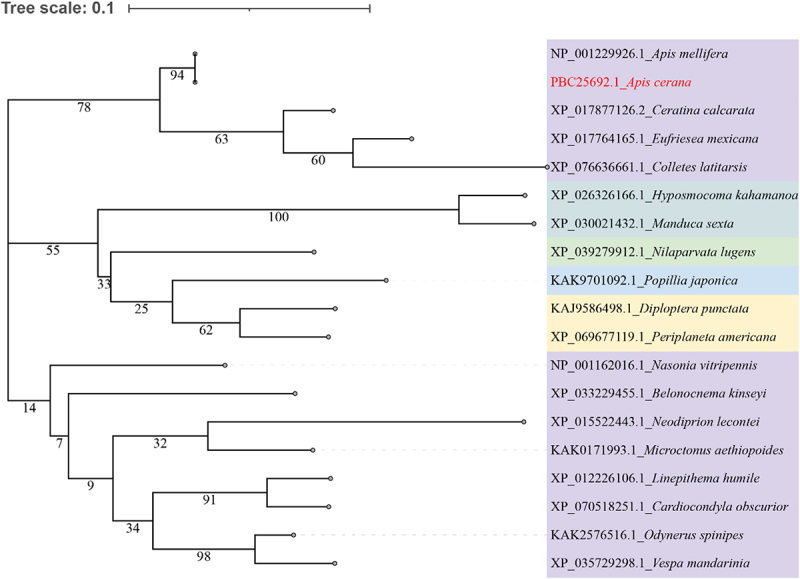
Phylogenetic analysis of *A. cerana* CTL5 and other insect CTLs. A total of 19 CTL amino acid sequences from other insects were used to construct a phylogenetic tree. This tree was constructed using the maximum likelihood method with 1000 bootstrap replicates. Bootstrap confidence values are shown at the internal nodes. The tree was visualized and optimized using the online iTOL platform, with *A. cerana* CTL5 highlighted in red.

The theoretical molecular weight of the mature AcCTL5 protein after removing the signal peptide was 23.07 kDa, and the isoelectric point was 8.22. To investigate the evolutionary relationships between *A. cerana* CLT5 and other insect CTLs, we retrieved amino acid sequences of other insect CTLs from GenBank and constructed a phylogenetic tree using the maximum likelihood method (Figure 2). The tree shows that AcCTL5 clusters with CTL5 of *A. mellifera* with high bootstrap value of 94, confirming their close evolutionary relationship. Other Hymenoptera CTL5 homologs were distributed in multiple lineages, while CTL5 sequences from Lepidoptera, Hemiptera, and other insect orders were positioned as outgroups. Comparison of the amino acid sequences and 3D structures of CTL5 proteins from *A. cerana* and *A. mellifera* revealed completely identical results (Supplementary Figure S1).

### Analysis of tissue expression profile of AcCTL5 in A. cerana

**Figure 3.**
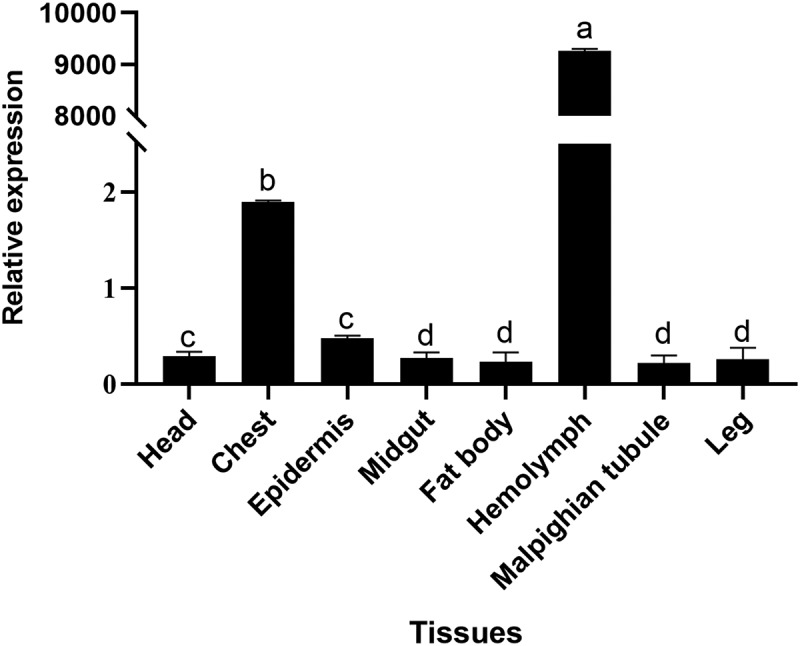
Expression of *AcCTL5* mRNA in different tissues of *A. cerana*. Data were presented as mean ± SD. Statistical significance was determined by one-way ANOVA followed by Tukey’s HSD post-hoc test. Different letters (a, b) indicate a significant difference between groups (*p* < 0.05); the same letter indicates no significant difference between groups.

The expression level of the *AcCTL5* gene in different tissues of healthy adult *A. cerana* was analyzed using quantitative real-time PCR (RT-qPCR). The results showed that there were differences in the expression levels of the *AcCTL5* gene among various tissues (Figure 3): the expression level was the highest in hemolymph, followed by the chest, epidermis, and head, while the expression level was the lowest in the malpighian tubule.

### Recombinant AcCTL5 can be effectively expressed and purified from E. coli

**Figure 4.**
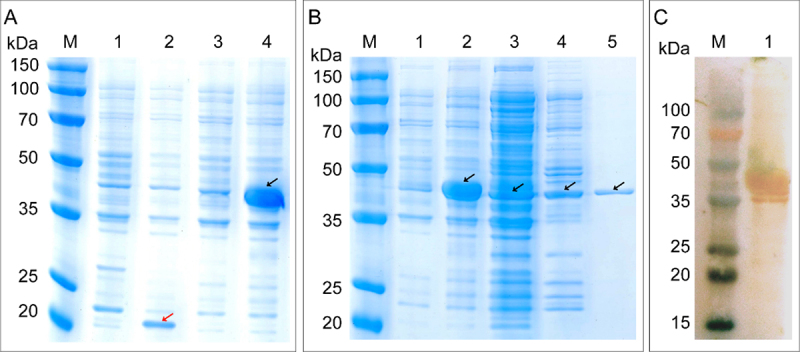
Inducible expression of recombinant AcCTL5 protein. (A) SDS-PAGE analysis of rAcCTL5. Lane M: protein molecular weight markers; lane 1: lysate of *E.Coli* carrying empty pET32 without IPTG induction; lane 2: lysate of *E.Coli* carrying empty pET32 induced by IPTG; lane 3: without IPTG-induced bacterial pellet carrying pET32-AcCTL5; lane 4: IPTG-induced bacterial pellet carrying pET32-AcCTL5. (B): Soluble rAcCTL5 protein purification; lane M: protein molecular weight markers; lane 1: bacterial pellet carrying pET32-AcCTL5 vector without IPTG induction; lane 2: bacterial pellet carrying pET32-AcCTL5 vector induced by IPTG; lane 3: bacterial supernatant carrying pET32-AcCTL5 vector induced by IPTG; lane 4: eluted with 25 mM imidazole, more bands; lane 5: eluted with 50 mM imidazole, bands single. (C) Western blotting analysis of anti-AcCTL polyclonal mouse antibody (1:200) and rAcCTL5 protein. The protein expressed by the pET32 vector is 20 kDa, indicated by a red arrow. The protein expressed by pET32-AcCTL5 is 43 kDa, indicated by a black arrow.

The pET32-AcCTL5 plasmid was transformed into *E.coli* Rosetta (DE3) competent cells. IPTG was used to induce the bacteria carrying the recombinant plasmid to produce the recombinant protein (pET32-AcCTL5). As shown in Figure 4(A), rAcCTL5 protein was successfully expressed in the supernatant of *E. coli* BL21 (DE3). Compared with the negative control pET32 vector (Figure 4(A), lanes 1 and 2), a distinct rAcCTL5 band was obtained by SDS-PAGE separation (Figure 4(A), lanes 4), and the molecular mass of pET32-AcCTL5 is 43 kDa, which is consistent with the predicted molecular weight of 23 kDa for AcCTL5, since the pET32 vector is 20 kDa (Figure 4(A), lane 2). rAcCTL5 was successfully purified from the supernatant by using Ni-NTA superflow cartridges (Figure 4(B)). The purified soluble protein was used for polyclonal antibody preparation, and subsequent functional analysis. Further, the successful expression of rAcCTL5 was verified by western blotting (Figure 4(C), lane 1). These results indicated that AcCTL5 was successfully expressed in vitro.

### Recombinant AcCTL5 exhibits specific binding to pathogen cell wall saccharides

**Figure 5.**
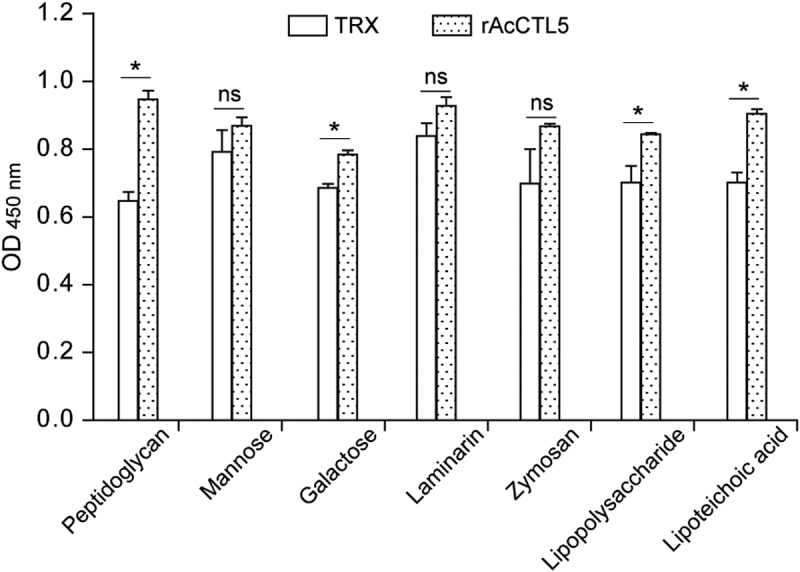
Carbohydrate binding specificity of rAcCTL5. Data are presented as mean ± SD. Comparisons between two groups were analyzed by Student’s t‑test. * means significant difference (*p* < 0.05); ns indicates no significant difference (*p* > 0.05).

To further determine which substances on the surface of the pathogen rAcCTL5 specifically binds, a binding experiment between the recombinant protein and cell wall sugar components was performed. The binding was evaluated by comparing the OD_4__50_ values of the rAcCTL5 group with those of the Trx-Tag control group, and statistical significance was analyzed by Student’s t-test. As shown in Figure 5, rAcCTL5 protein exhibited significantly higher binding to peptidoglycan, galactose, lipopolysaccharide, and lipoteichoic acid compared to the control (*p* < 0.05), whereas no significant difference was observed for mannose, laminarin, or zymosan (*p* > 0.05). These results indicate that rAcCTL5 is an active protein with carbohydrate‑binding ability and belongs to the galactose-type C-type lectins.

### Recombinant AcCTL5 can bind to N. ceranae

**Figure 6.**
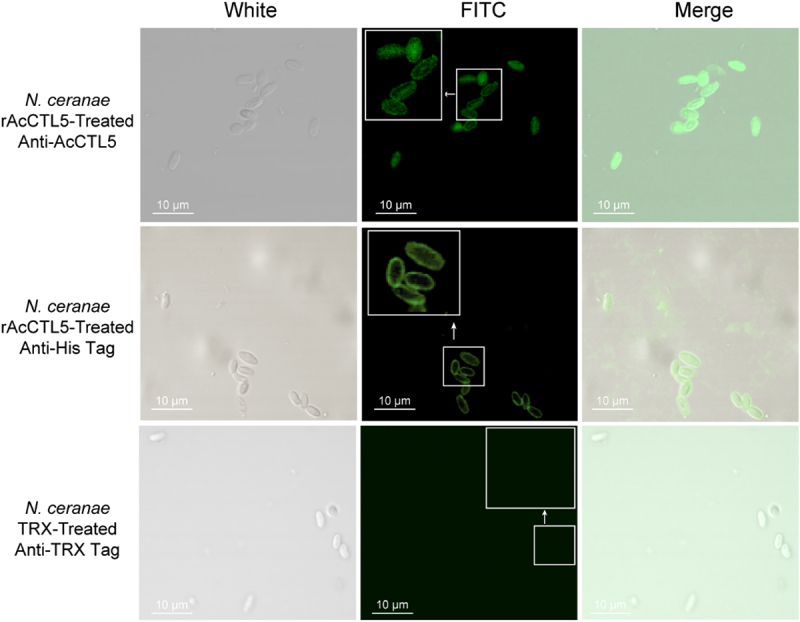
Binding of rAcCTL5 to surface substances of *N. ceranae*. The combination of rAcCTL5 with surface substances of *N. ceranae*; a green immunofluorescence signal appeared on the surface of *N. ceranae* treated with rAcCTL5. Trx tag antibody treatments served as negative controls, and the scale bar represents 10 μm.

To investigate whether rAcCTL5 binds to the surface of N. ceranae, indirect immunofluorescence assay (IFA) was conducted. When N. ceranae was treated with rAcCTL5 and subsequently incubated with anti-CTL5 antibody or anti-His tag antibody, prominent green fluorescence signals were clearly observed on the surface of N. ceranae under the FITC channel. In sharp contrast, the control group treated with thioredoxin (TRX, a control protein) and probed with anti-TRX tag antibody exhibited negligible fluorescence (Figure 6). Collectively, these results demonstrate that rAcCTL5, as a pattern recognition molecule in innate immunity, can participate in the recognition of surface substances of *N. ceranae* and specifically bind to them.

### Recombinant AcCTL5 can agglutinate N. ceranae

**Figure 7.**
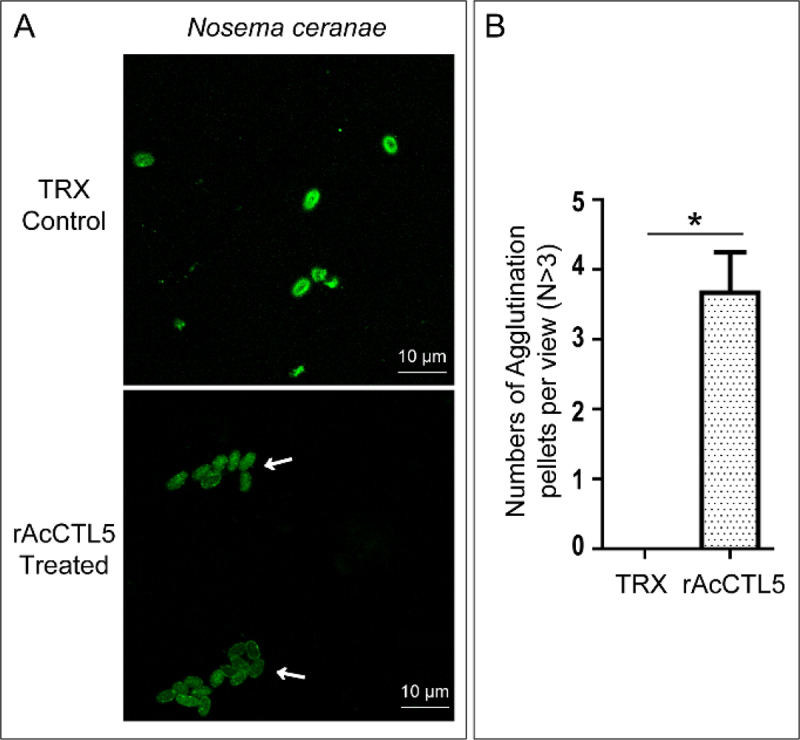
Analysis of the agglutination effect of rAcCTL5 protein on *N. ceranae*. Trx: pET32 empty tagged protein as a negative control. (A) The agglutination of rAcCTL5 on and *N. ceranae*. (B) Counting the number of agglutination groups of rAcCTL5 on *N. ceranae* under three different fields of view; Mean ± standard error of three parallel experiments; Comparisons between the rAcCTL5 group and the control group were analyzed by Student’s t‑test. * indicates *p* < 0.05.

To determine whether the binding activity of rAcCTL5 can induce microbial agglutination, fluorescently labeled *N. ceranae* was incubated with rAcCTL5 protein, and the agglutination activity was observed using by fluorescence microscope. The results showed that compared with the control group, the addition of rAcCTL5 had a stronger agglutination effect on *N. ceranae* (Figure 7(A)). To further clarify the agglutination effect of rAcCTL5 on *N. ceranae*, a histogram was established for analysis with the diameter of the formed colonies greater than 10 μm and the number of *N. ceranae* aggregates greater than 3 (Figure 7(B)). There was a significant difference between the experimental group and the control group, and rAcCTL5 had a significant agglutinating effect on *N. ceranae*.

### Recombinant AcCTL5 inhibits N. ceranae infection in honeybees

**Figure 8.**
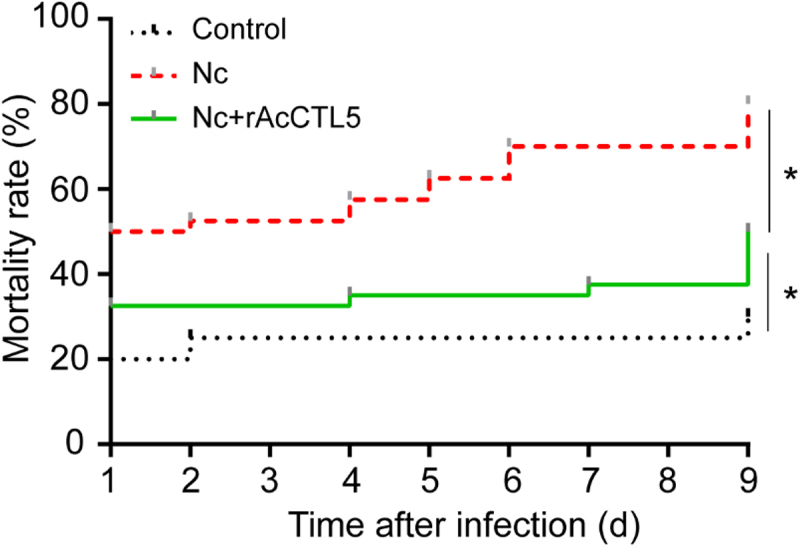
rAcCTL5 inhibits infection of honeybees by *N. ceranae*. Survival curves were estimated using the Kaplan‑Meier method and compared by the log‑rank test. Black: Negative control, healthy bees without any treatment; Red: Healthy bees fed *N. ceranae*; Green: *N. ceranae* and rAcCTL5 protein fed to healthy bees after incubation in vitro; * means significant difference (*p* < 0.05).

The rAcCTL5 protein was mixed with *N. ceranae* in vitro and the mixture was then fed to the bees. Results showed that compared with *N. ceranae* treated with rAcCTL5, the cumulative mortality of honeybees showed a significantly lower trend than that fed directly with *N. ceranae*; Compared with the control group, the cumulative mortality of *N. ceranae* treated with rAcCTL5 showed a slightly higher trend after feeding on bees. It shows that the presence of rAcCTL5 can effectively reduce the infection rate of bees (Figure 8).

### Recombinant AcCTL5 promotes hemolymph melanization level

**Figure 9.**
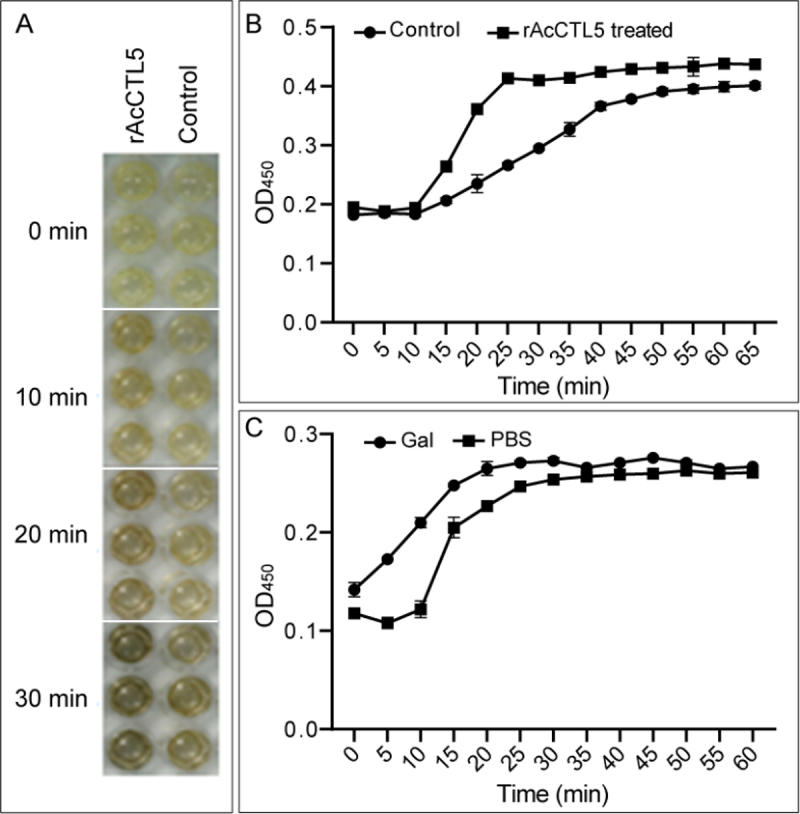
Activation of phenol oxidase by rAcCTL5 in honeybee. (A) The melanization phenomenon of haemolyph caused by rAcCTL5 in honeybees; (B) the values of OD of haemolyph under the condition of rAcCTL5 and TRX-tag, respectively; (C) the values of OD of haemolyph under the condition of Gal and PBS, respectively, in the presence of rAcCTL5.

After mixing rAcCTL5 protein with normal bee hemolymph, we observed that the melanization speed of bee hemolymph was significantly faster than that of the control hemolymph, indicating that rAcCTL5 protein can activate the melanization pathway of the hemolymph phenoloxidase cascade, causing hemolymph melanization (Figure 9(A,B)). In the presence of rAcCTL5 protein, the melanization effect was more obvious after adding an appropriate amount of galactose (Figure 9(C)). It shows that the existence of galactose can enhance the activity of C-type lectin, and then promote the melanization of hemolymph. This further confirms that bee CTL5 is a galactosyl-type lectin.

**Figure 10.**
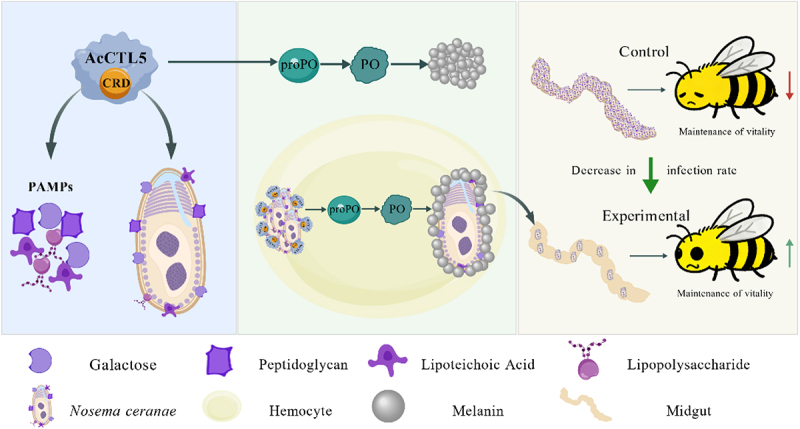
AcCTL5-mediated immune defense against *N. ceranae* in *A. cerana*. AcCTL5, a C-type lectin, recognizes various PAMPs on the surface of *N. ceranae* spores via its carbohydrate recognition domain (CRD), leading to spore agglutination. This recognition triggers dual immune responses: the activation of the prophenoloxidase (proPO) cascade, resulting in melanin synthesis (humoral immunity), and the encapsulation of spores by hemocytes, forming melanized nodules (cellular immunity). Collectively, these AcCTL5-mediated immune responses reduce the infection rate and protect the midgut integrity of honeybees. The figure was drawn using the online tool BioGDP [37].

In summary, this study clarifies that the C-type lectin AcCTL5, as a pattern recognition molecule in the innate immunity of *A. cerana*, can mediate agglutination by specifically binding to surface components of *N. ceranae*, while participating in the activation of the hemolymph melanization pathway, forming a coordinated defense mechanism of “recognition-agglutination-melanization” to effectively inhibit pathogen infection (Figure 10).

## Discussion

C-type lectins (CTLs) are key pattern recognition receptors in insect innate immunity, yet their roles against microsporidian pathogens such as *N. ceranae* remain poorly understood. In this study, we identified AcCTL5 from *A. cerana* and demonstrated that it contributes to anti-*N. ceranae* defense through agglutination and activation of hemolymph melanization. Sequence analysis revealed a single carbohydrate-recognition domain (CRD) with a galactose-binding QPD motif, a signal peptide, and no transmembrane region, indicating that AcCTL5 is a secreted galactose-type CTL. It shares 100% amino acid sequence identity with CTL5 of *A. mellifera*. This high conservation between two species with divergent life histories suggests that CTL5 has been maintained under persistent selective pressure, likely imposed by shared core pathogens such as microsporidia.

The mRNA of *AcCTL5* was most abundant in the hemolymph, consistent with the role of hemolymph as the central hub of insect innate immunity [38]. Once pathogens breach physical barriers and enter the hemocoel, hemolymph-borne CTL5 can recognize PAMPs through its CRD and initiate downstream immune responses [39]. In functional assays, rAcCTL5 induced hemolymph melanization, and this effect was enhanced by galactose. Melanization is a critical immune mechanism in insects [40], and multiple CTLs have been shown to modulate cellular immunity by promoting nodulation, encapsulation, prophenoloxidase activation, and melanization [23,41,42]. For instance, IML-10, HaCTL3, and the CTLD2 of MsIML-2 trigger encapsulation and melanization [28,40,43], while BmCTL5 in *B. mori* resides in the hemolymph, promotes nodule formation, and participates in the melanization cascade [44]. The combination of high hemolymph expression and galactose-dependent melanization therefore supports a model in which AcCTL5 acts as a surveillance molecule that couples sugar recognition to systemic melanization during *N. ceranae* infection.

Binding experiments further showed that rAcCTL5 recognizes a broad spectrum of microbial ligands—including PGN, LPS, LTA, and galactose—consistent with the specificity of its QPD motif. In the presence of Ca^2+^, rAcCTL5 strongly agglutinated *N. ceranae* spores and bound directly to the spore surface. Many insect CTLs mediate microbe agglutination: the CTL‑immulectins and CTL‑S of *M. sexta* agglutinate bacteria [45], HaCTL7 of *H. armigera* agglutinates both Gram-positive and Gram-negative bacteria [46], and mosquito CLSP2 agglutinates coccidioides in vitro [47], while certain CTLs also exhibit antifungal activity [30]. Given the galactose-binding QPD motif of AcCTL5, these properties position it as a Ca^2+^-dependent lectin that agglutinates *N. ceranae* spores by recognizing galactose-containing glycans on the spore surface. Importantly, rAcCTL5 reduced *N. ceranae* infection levels in *A. cerana*, indicating that this recognition-agglutination function translates into effective pathogen clearance in vivo and that AcCTL5 is likely an integral component of the immune resistance against microsporidia.

In conclusion, AcCTL5 functions as a galactose-specific, Ca^2+^-dependent PRR that recognizes *N. ceranae* via its CRD. It mediates spore agglutination to restrict pathogen dissemination and activates the hemolymph melanization cascade; this coordinated response both immobilizes the spores and exploits cytotoxic intermediates of melanization. This recognition-agglutination-melanization mode also provides a molecular basis for future disease control and molecular breeding strategies in honeybees.

## Supplementary Material

Supplementary file.docx

## Data Availability

The raw data referenced in this article are openly available in the Figshare repository under the CC0 license at https://doi.org/10.6084/m9.figshare.30507581 [48].
