# Supplementary material for: The C-type lectin AcCTL5 from Apis cerana mediates anti-Nosema ceranae defense via agglutination and hemolymph melanization
Source: Virulence. 2026 Jun 10;17(1):2687914. doi: 10.1080/21505594.2026.2687914 (PMC13274162; doi:10.1080/21505594.2026.2687914)
Supplement: Supplementary file.docx [file KVIR_A_2687914_SM4835.docx]

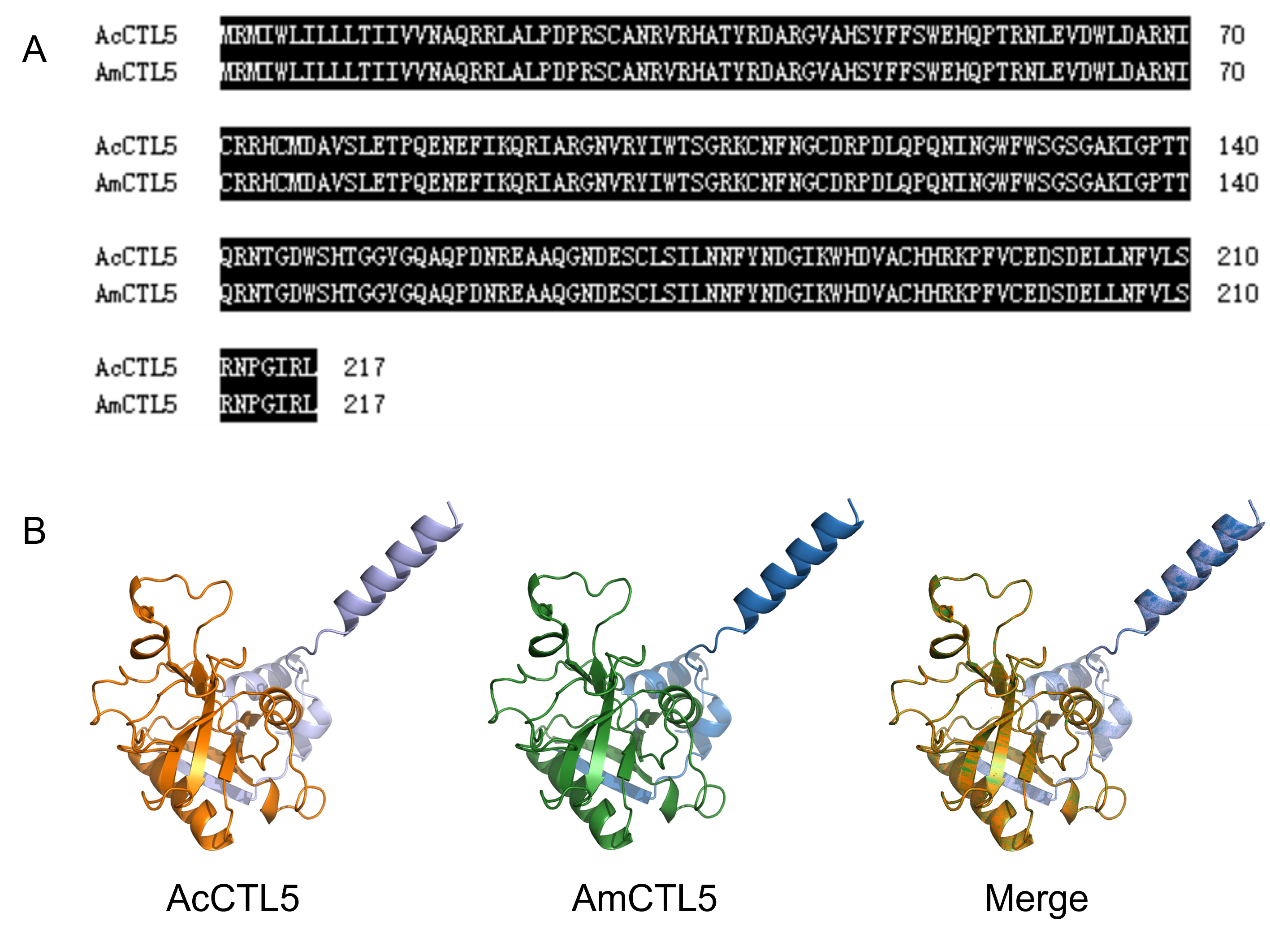


**Supplementary Figure 1.** Sequence alignment results of CTL5 from *A. cerana* and *A. mellifera*. (A) Amino acid sequence alignment of CTL5 from *A. cerana* and *A. mellifera* (B) 3D structure alignment of CTL5 from *A. cerana* and *A. mellifera*, where the Carbohydrate Recognition Domain (CRD) of *A. cerana* is shown in orange and that of *A. mellifera* is shown in green.
